# An Overview of Finnish Maternal Health Care As a Potential Model for Decreasing Maternal Mortality in the United States

**DOI:** 10.1089/whr.2021.0001

**Published:** 2021-03-09

**Authors:** Alexandra Schmidt, Gloria Bachmann

**Affiliations:** ^1^ Rutgers Robert Wood Johnson Medical School, Piscataway, New Jersey, USA.; ^2^ Women's Health Institute, Rutgers Robert Wood Johnson Medical School, New Brunswick, New Jersey, USA.

**Keywords:** maternal mortality, maternal health, perinatal care

## Abstract

***Background:*** The United States ranks poorly when compared with other developed nations with regard to its maternal mortality ratio (MMR), defined as the number of maternal deaths per 100,000 live births. Meanwhile, Finland consistently ranks as one of the safest places to be pregnant and give birth. The U.S. MMR more than doubled between 1987 and 2016, increasing from 7.2 deaths per 100,000 to 16.9, and has continued to increase. The Finnish MMR in 2017 was reported as 3 deaths per 100,000 live births, compared with the United States' 19 deaths for that same year. This article provides a comprehensive outline of Finland's structure of perinatal care, as well as a review of statistics concerning trends in the country's mortality and morbidity risk factors and a comparison with similar parameters in the United States.

***Methods:*** The Finnish maternal healthcare system was observed through the shadowing of healthcare providers during various pre- and postpartum patient encounters. Further discussion was supplemented by literature review.

***Results:*** Although trends among Finnish mothers for more than the past 30 years indicate increased prevalence of mortality and morbidity risk factors, including C-section rates, maternal mortality remains consistently low. Observational data depict the Finnish perinatal care system as a decentralized community-based network of primary health facilities that emphasizes both physical and psychosocial well-being in the care of its expectant mothers.

***Conclusion:*** We suggest that the Finnish perinatal system of care may provide a good template from which the United States can model future efforts to decrease maternal mortality.

## Introduction

Maternal mortality has become an important health priority in the United States as rates of maternal death nationwide continue to increase. The United States ranks poorly when compared with other developed nations with regard to its maternal mortality ratio (MMR), defined as the number of maternal deaths per 100,000 live births.^1^ Specifically, the U.S. MMR more than doubled in the 30 years between 1987 and 2016, increasing from 7.2 deaths to 16.9 deaths per 100,000.^2^ The United States is often described as the only developed nation whose MMR has consistently trended upward over the past few decades. Current discussion largely attributes this to the country's high rates of cesarean section delivery, but the issue is complex and multifactorial.^2^ Other important considerations include rates of comorbidities, racial disparity, and access to adequate prenatal care. It is imperative that we study other developed nations with significantly lower incidence of maternal death to better understand the gaps in our own maternal care system.

The Finnish MMR in 2017 was reported as 3 deaths per 100,00 live births, compared with the United States' 19 deaths per 100,000 live births for that same year.^3^ Finland consistently ranks as one of the safest places to be pregnant and to give birth and is recognized for its innovations in maternal care. One such innovation is the “baby box,” a government social welfare program first introduced in 1937 for low-income mothers to curb high infant and maternal mortality in that demographic.^4,5^ This basic cardboard package, which is still distributed today, was filled with essentials for early life, ranging from diapers and baby bottles to a thin mat that allows the box to serve as a crib. The contents of the package have evolved with time; fabrics originally provided for moms to sew their babies' clothes were replaced with premade clothing in the 1970s as more women entered the workforce, and bottles were removed as recently as the early 2000s to promote breastfeeding.^5^ The box today also provides toys, as well as picture books to promote reading in the home. The program was so successful that it was later expanded to include all women in the country, with the added eligibility requirement of receiving prenatal care by the fourth month of pregnancy.^5^ With this integration into the country's maternal health care system, the baby box has become one of the interventions contributing to Finland's extremely low maternal and infant mortality rates by promoting early engagement with prenatal care services.

Another important contributor to Finland's success is the maternal health care system in which Finnish women receive their care. The current Finnish system has been described as a decentralized network of primary health centers that serve local communities.^6^ This differs greatly from the infrastructure of the United States, which comprises medical centers and clinics, rather than a community-based health care network. This obvious intervention may serve as a model for countries with higher maternal mortality rates such as the United States. This article reviews the structure of the Finnish system of care and how its adoption in the United States may result in safer care to expectant mothers.

## Methods

In addition to reviewing reports regarding maternity care in both Finland and the United States published by Finland's National Institute for Health and Welfare (THL), the Centers for Disease Control (CDC), and the World Health Organization (WHO), 30 low-risk pre- and postpartum appointments were observed in the local maternal clinic of Hervanta, Tampere, Finland, and 5 high-risk appointments were observed at Tampere University Hospital. Additional interviews with six public health nurses, four doctors, and one midwife were obtained across both locations.

## Results

### General structure of care

The Finnish maternal health care system comprises community-based clinics, called “äitiysneuvola,” which are run by a staff of multiple terveydenhoitajat (“public health nurses”) and one or two general physicians. Terveydenhoitajat are nurses who have completed additional training to specialize in perinatal and pediatric care. Unless otherwise noted, the term “nurse” in this article will be used to refer to terveydenhoitajat. Physicians at the clinic have completed general medical training of 6 years duration. Overall clinic structure can vary between locations, with many working in conjunction with other medical specialty offices. For example, the clinic in which observational data for this project were collected also housed a dentist, a psychologist, and a speech language therapist. Ultimately, all clinics provide comparable services across the nation, free of charge at time of service.

A structured schedule of prenatal appointments based on recommendations from the national Finnish THL is consistent throughout the country, not varying due to geography, socioeconomics, or providers. The template of care commences when a woman, after confirming pregnancy by an at-home test, reaches out to her local clinic to schedule an appointment. Women are then paired with a specific clinic nurse as their primary care provider for the duration of the pregnancy. Their first appointment with this nurse is scheduled for 8–10 weeks of gestation and is allocated 90 minutes, which is the time specifically provided for both patient and provider to get to know one another, in addition to a general physical examination. It is not unusual for the baby's father to be present for this appointment as well. If a woman has previously had children, she will be paired with the same nurse who oversaw her preceding pregnancies, if possible. In these cases, the full 90 minutes for the first appointment may not be required.

Subsequent prenatal visits are conducted by this same nurse and range from 20 to 90 minutes (Table 1). These appointments vary in scope. Some encompass basic check-ups of general measures of well-being, that is, blood pressure, fetal heartbeat, and hemoglobin levels, whereas others are apportioned more time to also discuss psychosocial issues, such as smoking cessation, fears surrounding childbirth, and expected social support for the postnatal period. Normally only two appointments, at 9–10 and 35–36 weeks of gestation, are allocated for assessment by a clinic physician. These appointments generally last 20 minutes and include a gynecological examination.

**Table 1.** Program for Periodic Maternal Health Check-Ups, 2018

Appointment	Time allotted	Provider
Phone call	—	Nurse
8–10 weeks	90 minutes	Nurse
14–18 weeks	20 minutes	Doctor
22–24 weeks	60 minutes	Nurse
26–28 weeks	45 minutes	Nurse
30–32 weeks	90 minutes	Nurse
35–36 weeks	20–40 minutes	Doctor
37–41 weeks	30 minutes	Nurse
Home visit^a^	60 minutes + travel	Nurse
Postpartum check-up^b^	20 minutes	Doctor

“Äitiyshuollon Määräaikaistarkastusten Ohjelma 2018.”

General overview of perinatal appointment scheduling in Finland.

^a^ The home visit usually occurs within 1 week of parturition at the mother's home.

^b^ The postpartum check-up occurs at the clinic, at ∼6 weeks postpartum.

The goal for all healthy pregnancies is vaginal birth. Parturition occurs at the local hospital and is attended by midwives, with physician involvement occurring only when conditions dictate their presence. Midwives may consult physicians but usually take care of procedures themselves, for example, induction of labor and/or administration of oxytocin. Some interventions require the presence of a physician; for example, the placement of an epidural breach and twin deliveries, and the use of vacuum extraction or forceps to assist in the birth. If no physician intervention is required, two midwives are present at the time of delivery.

After giving birth, patients are discharged from the hospital into the care of their local clinic system. Within a week of parturition, or 3 to 4 days after being sent home from the hospital, the same nurse who oversaw the pregnancy conducts a 60-minute home visit to assess both the new mother and baby's physical well-being. This includes measuring uterine size, checking the baby's weight gain, and providing help with breastfeeding, if required. The mother's emotional and mental status are also observed, with common topics of discussion involving her feelings toward the labor and delivery experience, methods of contraception to use in the postnatal period, and other aspects of psychosocial and sexual health. Occasionally, women opt to forego the home visit and choose to see their nurse in the clinic setting at this time instead.

The new mother and baby present to the clinic for a postnatal check-up with the physician 6 weeks after parturition. This marks the conclusion of the mother's care at the clinic. Babies remain patients of the clinic until they are 7 years old, at which time they begin primary education and the responsibility of their health care management is transferred to a comparable clinic system overseen by the local public schools.

### “High risk” pregnancy

The structure of care for high-risk pregnancies does not adhere to the timeline template as already outlined. Women who require a more intensive level of care than can be provided at the community clinics are referred to maternal “policlinics,” which are located in the hospital. These policlinics are staffed primarily by physicians who have undergone additional training to specialize in areas of care such as perinatology, endocrinology, and gynecological oncology. These are the same physicians who are consulted by midwives on the labor and delivery floor and who perform intrapartum procedures.

The proportion of visits at the community clinic versus the hospital policlinic for “high-risk” women is highly individual and determined on a case-by-case status. A woman who has experienced complications in a previous pregnancy may be referred to the policlinics from the start of her pregnancy. Another woman may present to the community clinic for a normal check-up, at which point concerning findings (*e.g.*, abnormal ultrasound results, pathological glucose test, and dangerously elevated blood pressure) prompt the community clinic staff to send her to the policlinic for follow-up. Note that the purpose of this article is not to delineate the precise protocols in place for all possible complications during pregnancy. Rather, the aim is to emphasize how the accepted standard of care is tailored to individuals as needed.

### Communication, education, and resources for pregnant women

Information from each appointment throughout pregnancy is noted in an äitiyskortti (“maternity card”). Traditionally, this was a physical paper fold-out card, but most municipalities have moved to online versions. All providers involved in the patient's care, as well as the patient themselves, can access the information contained in this account. Care providers use it to track important milestones and health data throughout pregnancy, whereas patients can use it to log their symptoms and jot down questions for their providers.

Pregnant women set up this iPana account before or during the first prenatal appointment. They are also given the cell phone number of their assigned nurse for direct contact. Women are encouraged to call if they have any questions or concerns, such as new symptoms. In the clinic audited for this project, the nurses have “call hour” every day from 12 to 12:30, but women can call at any time throughout the day if the need arises. During off hours, or for more urgent matters, women are directed to call the national health hotline, which is available as a medical resource for all, not just for pregnant women.

The community clinic provides group workshops every few months that address common concerns, ranging from breastfeeding to fears surrounding delivery. Many appointments throughout gestation have time set aside specifically for these discussions, and additional educational resources centered on many of these same topics can also be found online through a centralized government website. There is also the option to tour the delivery unit at the local hospital and speak with a midwife well before a patient's due date. This service is generally provided to women who have expressed fears regarding natural childbirth in an attempt to assuage reservations and to encourage attempting vaginal delivery.

### Mortality risk factors and other statistics

According to the WHO, the Finnish maternal mortality rate decreased from 6 per 100,000 live births in 2000 to 3 per 100,000 in 2017 and has remained consistent since.^3^ According to data published by Finland's THL that spans the 30 years from 1987 to 2017, it appears that many of the high-risk factors noted in the United States are also increasing in Finland. Analogous U.S. data are included for comparison.

National Finnish data demonstrate that the mean length of stay (LOS) in the hospital after birth was 2.7 days in 2017. This comes after a steady decrease in stay duration from an average of 6.6 days in 1987.^7^ Studies in the United States indicate a similar downward trend in the late 20th century, with LOS decreasing from 4.1 days in 1970 to 2.6 days in 1992.^8^ A more recent study indicated the average LOS in the United States ranged from 48 to 96 hours for the majority of obstetric patients.^9^

National Finnish data demonstrate a steady rise in the rate of cesarean section births.^7^ The overall C-section rate increased from 14.5% (1987) to 16.7% (2017), with the percentage of women requiring an urgent C-section increasing from 7.6% (2005) to 9.1% (2017). Within that same period of time, the proportion of women undergoing a planned C-section decreased from 7.8% (1995) to 6.8% (2017). In the United States, overall C-section rates have also increased since the late 20th century. A fivefold increase from 4.5% in 1965 to 22.7% in 1985 precluded a slower rise in surgical delivery that eventually peaked at 32.9% in 2009.^10,11^ The U.S. C-section rate has remained steady or decreased in the time since then, with a 0.1% increase from 31.9% (2016) to 32.0% (2017) the first increase recorded in this period. The rate ticked downward again in 2018 back to 31.9%.^11^

Rates of most other intra- and postpartum procedures in Finland also increased during this window.^7^ Induction of labor more than doubled between 1990 and 2017, from 14.0% to 28.9%. The proportion of women receiving any kind of pain relief during labor rose from 78.0% in 1995 to 92.2% in 2017, with all subsets of pain relief options increasing in frequency. For example, from 1987 to 2017, the proportion of women receiving an epidural increased from 8.2% to 50%, paracervical blocks increased from 12.0% to 15.8%, and pudendal blocks increased from 0.2% to 12.1%. The use of vacuum extraction to assist in delivery increased from 3.5% in 1987 to 9.3% in 2017. Conversely, episiotomy rates decreased from 47.1% (1995) to 20.1% (2017).

Comparable data within the United States show similar trends. Rates of induction of labor increased from 9.6% in 1990 to 27.1% in 2018.^11,12^ Use of epidural pain relief also appear to be increasing: 68.1% of nulliparous women and 51.3% multiparous women received an epidural in 2008, with 78.8% nulliparous and 64.4% multiparous women receiving an epidural in 2013.^13,14^ Episiotomy rates decreased from 60.9% in 1979 to 24.5% in 2004, and followed this downward trajectory further to 7.8% in 2017.^15,16^

Furthermore, national Finnish data also reflect a concomitant increase in maternal health risk factors.^7^ The data indicate that in the 10 years between 2007 and 2017, the average body mass index (BMI) of women before pregnancy marginally increased from 24.2 to 24.8 kg/m^2^. Over the same time period, however, the percentage of women characterized as obese before pregnancy (BMI >30 kg/m^2^) increased from 11.2% to 14.4%. Correlate rates of gestational diabetes rose from 6.4% to 15.6%, with 19% of women in 2017 having had pathological results of a glucose tolerance test some time during pregnancy, up from 9.5% in 2007. Another important risk factor, tobacco use during pregnancy, fluctuated over this time period but ultimately decreased from 15.5% of pregnant Finnish women in 1987 to 12.5% in 2017.

The percentage of women in the United States who were overweight or obese (BMI >25 kg/m^2^) before pregnancy was 54.7% in 2018.^11^ This followed a 2% increase in overweight status and an 8% increase in obese status before pregnancy during the years 2011–2015.^17^ The rate of gestational diabetes in 2018 was 6.7%, up from 3.71% in 2000.^11,18^ Smoking during pregnancy decreased from 13.3% in 2000 to 6.9% in 2017.^19,20^

## Discussion

The United States is actively addressing the high rate of maternal mortality with changes to policies and standards of care. One major change includes a coordinated effort to decrease the total number of cesarean section deliveries.^21,22^ This is an important strategy in maternal risk reduction, as the WHO published a study in 2015 indicating that a C-section rate >10% no longer has a positive benefit toward reducing maternal mortality.^23^ These efforts also mirror the noninvasive outlook inherent to the Finnish system, as already outlined. Interestingly, C-section rates in Finland demonstrate a steady increase year over year since 1987 and have been above the suggested 10% rate for >30 years, all while the country's maternal mortality has remained consistently low.^3,7^ This trend in Finland also is reflected in a general increase in intrapartum procedures overall. In addition, trends in known health risk factors such as prepregnancy obesity are comparable between the two countries, with other important risks such as tobacco use and gestational diabetes actually higher in Finland than in the United States. These data suggest that it is the structural differences between the two health care systems that explain the discrepancies between maternal outcomes between Finland and the United States rather than solely differences in maternal risk factors.

The Finnish data describe a decentralized community-based network of primary health facilities tasked with educating and supporting women through all aspects of pregnancy. Importantly, nurses function as primary care providers rather than physicians. Other notable differences between Finland and the United States include the regular presence of a midwife instead of a physician at parturition and a routine postnatal home visit. The Finnish system also appears to provide extended time to establish an interpersonal relationship between patient and provider and to place more emphasis on the psychosocial aspects of pregnancy. Appointments are routinely allotted up to 90 minutes to ensure there is adequate time to discuss all aspects of a patient's experience. A nurse-directed postnatal home visit is performed not only to ensure the physical health of both mother and infant, but to assess emotional and mental well-being as well.

The Finnish system serves as a proven example of how a perinatal care system may be structured to ensure positive maternal outcomes. However, there are numerous limitations when comparing Finland and the United States. Engagement with prenatal care services differs between the two countries. Over 99% of Finnish women engage with the maternity clinic services as already described.^7,24^ A standard clinical performance measure commonly used in the United States to assess the quality of maternal health is the proportion of women who initiate prenatal care in their first trimester, and only 77.1% of U.S. women met this criterion in 2016.^25^ In addition, for all women in the United States who received any prenatal care, only 75% received at least adequate care.^25^ These differences in receipt of prenatal care may be due to various barriers to care in the United States, ranging from health care costs to lack of transportation.^26,27^ Universal health care and a robust public transportation system in Finland seem to largely eliminate these barriers for Finnish women, as evidenced by the high proportion of women receiving prenatal care. As such, accessibility to and receipt of prenatal care remain an important consideration when making comparisons between Finland and the United States.

The difference in population size between the two countries is also significant. With a population of >328 million, the United States has ∼60 times the number of people as Finland, which has a population of roughly 5.5 million.^28,29^ Notable differences in population demographics also exist. Finland is a largely homogeneous majority white country, with only relatively recent influxes of immigrants and refugees contributing to a small but growing ethnic minority population.^28^ Conversely, the United States is very diverse: ∼60% of the population is non-Hispanic white, 18% Hispanic or Latino, 13% black or of African descent, and 6% Asian, with the remaining proportion made up of native populations and people of multiple races.^29^

It is important to note that maternal mortality rates differ regarding these population demographics. For example, the average MMR for non-Hispanic white women in the United States between 2007 and 2016 was 12.7, whereas in that same time period, the average MMR for non-Hispanic black women was 40.8.^30^ Finland does not collect data on racial demographics, and thus there are no analogous stratifications of Finnish maternal mortality by race. However, it is notable that the Finnish maternal mortality rate is lower than even the lowest rates of maternal death in the United States when broken down by race.^30^ This suggests that the Finnish system may still provide an adequate comparative system to the United States despite differences in demographic factors, but studies that examine the impact of race on Finnish maternal outcomes would greatly add to this discussion.

Limitations of this review include the scope of this observational study, as well as inconsistencies in data collection between the two countries. Although only one local clinic and one high-risk hospital clinic were audited, these results can be applied to all sites of Finnish maternity care due to the adherence of these clinics to a national template of care. Furthermore, analogous statistics over similar time periods have been provided despite inconsistencies in reporting for different risk factors and procedures in the United States and Finland. For example, the 2017 Finnish statistics only report prepregnancy obesity (BMI >30 kg/m^2^), whereas the United States reports overweight status (BMI >25 kg/m^2^) and then delineates for trends in prepregnancy obesity and overweight. We believe that the data are presented in such a way for this and other risk factors that comparisons may be easily extrapolated between the two countries.

It is clear that increasing maternal mortality in the United States is a complex issue. Further research should be conducted in targeted geographic areas to determine whether a structure of care similar to that of Finland would be as successful if implemented in the United States, especially in areas of high maternal mortality. This may mean incorporating certain aspects of the Finnish system, such as nurse-directed home visits or increased midwife involvement, into pre-existing templates of care, rather than adopting it over completely. There are current initiatives within the United States with unique approaches to perinatal care that could provide avenues for this incorporation, as well. One such initiative, CenteringPregnancy, employs a group care model, with recent literature suggesting physical health and psychosocial benefits for both moms and babies.^31^ Many aspects of CenteringPregnancy are actually similar to aspects of the Finnish system, including 90-minute appointments and the involvement of nurses and midwives.^32,33^ Another, more logistical, initiative includes Medicaid expansions across the country, which has improved accessibility to perinatal care by guaranteeing coverage for pregnancy. In at least one state, this coverage even includes CenteringPregnancy.^34^ This demonstrates that alternative approaches to maternal health care are becoming more widely accepted, which potentially decreases logistic barriers to their implementation.

Overall, the Finnish system exemplifies the importance of utilizing a psychosocial model in treating expectant mothers. We believe that future research will demonstrate that efforts that aim to decrease maternal death must be multifactorial and interdisciplinary in nature. Unique initiatives that emphasize a holistic approach to perinatal care such as CenteringPregnancy demonstrate that the adoption of a care structure similar to Finland's structure is within the realm of feasibility and may very well prove beneficial toward improving maternal outcomes in the United States. We, therefore, believe it is worth exploring how adaptations from Finland's system of perinatal care could be incorporated into either pre-existing or future efforts at reducing maternal death in the United States.

## Abbreviations Used

BMI: body mass index
CDC: Centers for Disease Control
LOS: length of stay
MMR: maternal mortality ratio
WHO: World Health Organization
